# Predicting review helpfulness in the omnichannel retailing context: An elaboration likelihood model perspective

**DOI:** 10.3389/fpsyg.2022.958386

**Published:** 2022-09-13

**Authors:** Zhebin Zhang, Haiyin Jiang, Chuanmei Zhou, Jingyi Zheng, Shuiqing Yang

**Affiliations:** Zhejiang University of Finance and Economics, Hangzhou, Zhejiang, China

**Keywords:** review helpfulness, omnichannel retailing, review label, review context, review label-content relevance

## Abstract

As increasingly retail enterprises have adopted the omnichannel retailing strategy, both online-generated and offline-generated reviews should be considered to better understand the helpfulness of online reviews in the omnichannel retailing context. Drawing on the Elaboration Likelihood Model, the present study attempts to examine the impacts of review label volume, review content length, and review label-content relevance on review helpfulness in the omnichannel retailing context. The empirical data of 2,822 product reviews were collected from Suning.com. The results of Negative Binomial Regression showed that both central cue (review label-content relevance) and peripheral cue (review content length) positively affect review helpfulness. Specifically, the positive effect of review content length on review helpfulness will be stronger when the online review is submitted from an omnichannel retailer’s online store. On the contrary, the positive effect of review label-content relevance on review helpfulness will be weaker when the online review is generated from an omnichannel retailer’s online channel.

## Introduction

Online customer reviews play an increasingly important role in online commerce which has attracted scholars and practitioners’ attention around the world (Yang et al., 2021; Li et al., 2022; Wu et al., 2022). Extensive numbers of product reviews provide rich information for consumers which can help them decrease the degree of uncertainty and risk (Zhu and Zhang, 2010; Yang et al., 2019). However, a large number of reviews may cause significant information overload and high search costs for the reader (Siering et al., 2018). These problems may confuse customers about product quality and make it difficult for them to form purchase decisions (Wu et al., 2022). To address these problems, many e-commerce websites resort to product review systems that enable customers to vote on whether a review is helpful or not (Hong et al., 2017; Malik and Hussain, 2017; Yang et al., 2019). Reviews with higher voting rates will have greater weight in potential customers’ purchase decisions (Yang et al., 2019, 2021; Li et al., 2022). Therefore, researchers and practitioners must understand the factors that influence review helpfulness.

Many scholars have examined the factors that affect review helpfulness (Yang et al., 2019, 2021; Aghakhani et al., 2020; Wu et al., 2022). Factors such as review rating (Aghakhani et al., 2020), review content (Qazi et al., 2016), review length (Hong et al., 2017), and review image (Karimi and Wang, 2017) were identified as influencers of review helpfulness. In recent years, review labels aggregated for each review content have been adopted by a huge number of online review systems providers to reduce customers’ cognitive efforts for writing product reviews (Yi et al., 2017). However, despite its importance, the underlying mechanisms of how the relationship between review labels and related content influences the usefulness of reviews is still unclear. Therefore, it is interesting to systematically explore the influences of review labels and content on review helpfulness.

In addition, existing studies have mainly examined the factors that affect review helpfulness from a single-channel perspective (Ghose and Ipeirotis, 2011; Filieri et al., 2017; Yang et al., 2021). However, this single-channel context research cannot fully capture the influencers of review helpfulness in the omnichannel context. With the development of mobile technologies, many retailers have embraced an omnichannel strategy to offer customers a seamless shopping environment that allows them to shop across channels anywhere and anytime (von Briel, 2018; Yang et al., 2019). Omnichannel retailing refers to “the integration of retail channels like stores, online, and mobile into a single, seamless customer experience” (von Briel, 2018, p. 1). In the omnichannel retailing environment, online reviews can be submitted by consumers from either online or offline channels (Xiang et al., 2018; Yang et al., 2019). The online-based reviews are submitted by customers who have online purchasing experience in the digital store of an omnichannel retailer but without the omnichannel retailer’s online store shopping experience (Yang et al., 2019). On the contrary, offline-based reviews are generated by customers who have shopping experience in the physical stores of an omnichannel retailer (Li et al., 2019). Offline-based reviews may have a greater influence on the review readers than the online-based reviews because the former reflect customers’ omnichannel shopping experiences (Yang et al., 2019). Thus, it is important for us to explore whether the reviews submitted from different channels will exert different influences on the review helpfulness in the omnichannel shopping context.

Therefore, this study applies the Elaboration Likelihood Model (ELM) to theoretically analyze the factors that affect review helpfulness in the omnichannel retailing context. Specifically, this study examines the following questions: (1) How do the peripheral cues (e.g., review label volume, review content length) and central cues (e.g., label-review relevance) affect review helpfulness? (2) What are the different effects of peripheral cues and central cues on online review usefulness, when reviews are submitted from different review channels in an omnichannel retailing context? This study has several contributions to the extant literature. First, different from many previous studies which analyze factors that affect review helpfulness from a single channel, this study explores the antecedent variables of review helpfulness in an emerging omnichannel retailing environment. Second, this study validates the Elaboration Likelihood Model in an omnichannel retailing environment and develops a research model to explain review helpfulness by considering both the peripheral route and central route. Finally, unlike extant studies which tended to examine the impacts of review content-related factors on review helpfulness, this study explores the influences of the volume of review labels, as well as the review label-content relevance on review helpfulness.

The remainder of this study is organized as follows. First, this study presents the literature review of review helpfulness. Second, this study presents the research model and hypothesis. Next, this study explains the data collection procedure and the meaning of different variables. In the following section, this study summarizes the result of our results and concludes the study by discussing the contributions, limitations, and future directions of the study.

## Theoretical background

### Review helpfulness

Review helpfulness refers to the degree to which consumers believe that reviews help make purchase decisions (Mudambi and Schuff, 2012; Malik and Hussain, 2018; Yang et al., 2021). Various factors, such as review length (Chua and Banerjee, 2015), review content (Qazi et al., 2016), and review length (Hong et al., 2017), have been identified as the influencers of review helpfulness. For instance, extant studies have found that longer online reviews contain more product details that are helpful to customers, which validates the relationship between review length and review helpfulness (Chua and Banerjee, 2015; Yang et al., 2021). In recent years, review labels have been widely used on major comment websites (Bao et al., 2021). Review labels are user-generated review “tags” which are aggregated for each review and semantically describe various characteristics of a review (Yi et al., 2017; Bao et al., 2021). The review label makes it easier for customers to identify different types of information about products without reading lengthy comments. For instance, review labels can increase the diagnosticity of a review, thus improving the helpfulness of the review. Previous studies on review labels focused on issues, such as the growth patterns of labels (Golder and Huberman, 2006), consumer’s incentives for generating labels (Ames and Naaman, 2007), information organization efficiency using labels (Richard et al., 2009), and the spread effect of labels in social networks (Choi et al., 2015). Extant studies suggested that review labels can effectively activate consumers’ goal-related cognitive processing (Larson and Czerwinski, 1998; Katz and Byrne, 2003; Bao et al., 2021). However, the extant literature has yet to explicitly identify the impacts of review label volume and the relevance between review label and review content on review helpfulness.

### Elaboration likelihood model

The Elaboration Likelihood Model (ELM) proposed that both central routes and peripheral routes will persuade message recipients (Aghakhani et al., 2020; Ruiz-Mafe et al., 2020; Yang et al., 2021). According to the ELM theory, the process of individual persuasion can be induced by the peripheral route based on the attractiveness of the message source or the central route based on the strength of the argument in a specific message (Petty and Cacioppo, 1986; Cyr et al., 2018). The distinction between the two routes depends on the elaboration likelihood of the individuals (Cyr et al., 2018). In the high level of elaboration likelihood conditions, people tend to follow a central route which includes resorting to rational cognitive factors and attempting to evaluate new information logically. In the low level of elaboration likelihood conditions, people tend to follow a peripheral route which includes emotional factors by connecting the product with their attitude.

The ELM has been applied to explain and understand how central and peripheral routes affect the review helpfulness (Baek et al., 2014; Aghakhani et al., 2020; Yang et al., 2021). For instance, Aghakhani et al. (2020) found that central cues can be conceptualized by the factors related to the review text, while peripheral cues can be conceptualized by heuristic factors related to a review. They found that the consistency of review text and its review rating has a positive effect on review helpfulness. Baek et al. (2014) found that both peripheral cues (e.g., review rating and reviewer’s credibility) and central cues (e.g., content of reviews) can influence review helpfulness. Following previous studies (Bhattacherjee and Sanford, 2006; Filieri et al., 2018a; Yang et al., 2021), this study conceptualized review label volume and review content length as the peripheral cues, while review label-content relevance was conceptualized as the central cue. Petty et al. (1981) showed that personal involvement can be a motivational factor in ELM. This is important in the omnichannel environment because reviews from an omnichannel environment contain details about the product which need customers to touch and feel, so it has a high level of personal involvement (Petty et al., 1981; Yang et al., 2021). Thus, it is crucial for researchers to explore whether the reviews submitted from different channels will have different influences on the impacts of the peripheral and central cues to review helpfulness.

### Omnichannel retailing

Different from multichannel retailing, which operates channels separately to fulfill different customer needs (Ailawadi and Farris, 2017), omnichannel retailing aims to integrate separate online and offline channels to provide a seamless customer experience (von Briel, 2018; Li and Gong, 2022). In the omnichannel retailing context, customers can adopt different channels for product search, product purchase, and after-sale service (Li and Gong, 2022). Take Suning.com as an example, where mobile commerce customers can experience virtual shopping in an omnichannel retailer’s digital stores, at the same time, they can also have a shopping experience in the physical store with the help of offline retailer’s assistance (Li et al., 2019).

In an omnichannel retailer’s online channels, potential customers may consider the reviews submitted from an omnichannel retailer’s offline channel to be more helpful than that submitted from its corresponding online channel (Yang et al., 2019). Indeed, in an omnichannel retailer’s offline channels, customers can touch and feel products, and the reviews submitted by them may more likely to reduce the risk perceived by potential customers, which may enhance the review helpfulness (Huang et al., 2017). In academia, extant studies have explored the antecedents of review helpfulness in the omnichannel retailing context. For instance, Yang et al. (2019) studied the impact of review-related and reviewer-related on review helpfulness and found that when the review is submitted from offline channels, the positive impact of the reviewer’s real name on review helpfulness is stronger, while the positive impact of reviewer expertise on review helpfulness is weaker. Chatterjee and Kumar (2017) compared functional and expressive products within different product life length. They found that consumers prefer to durable expressive product through omnichannel retailing. Thus, it is interesting to examine the role of the online-generated and offline-generated reviews in sharping the peripheral and central routes on review helpfulness in the omnichannel retailing context.

## Research model and hypothesis development

**FIGURE 1:**
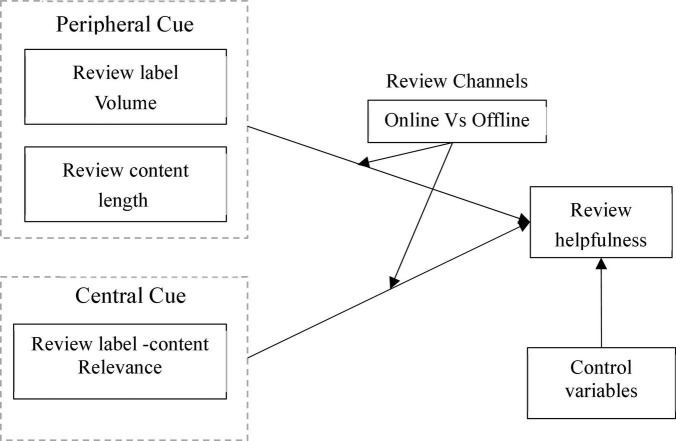
Research framework.

The proposed research model was developed based on the ELM theory which reflected the impacts of review label volume, review content length, and label-review relevance on review helpfulness in the omnichannel retailing context. The research model is shown in Figure 1.

### Elaboration likelihood model’s peripheral cue: Review label volume and review content length

Labels on a review column display a wide range of product features and each label leads consumers to an information patch (Yi et al., 2017). These features that reflect consumers’ terminology are created by collaborative tagging systems, and hence readers are more likely to understand them against their requirements (Golder and Huberman, 2006). In fact, some highly popular labels, which are frequently used by consumers to describe product characteristics, often represent precise descriptions of the protruding product features based on consumer groups (Yi et al., 2017). These labels can effectively activate consumers’ cognitive processes related to product reviews (Larson and Czerwinski, 1998; Katz and Byrne, 2003). Based on the ELM theory, the peripheral cue reflected by review label volume will positively influence review helpfulness (Filieri et al., 2018a; Yang et al., 2021). The more review label volume, the more likely the online review will be perceived as more helpful (Yi et al., 2017). Therefore, based on the extant studies (Larson and Czerwinski, 1998; Katz and Byrne, 2003; Yang et al., 2021), we can hypothesize that,

**H1a.** Review label volume will positively affect review helpfulness.

Review content length is defined as the number of words in an online review, which is considered one of the most basic variables to predict helpfulness an online review (Chua and Banerjee, 2015; Hong et al., 2017; Malik and Hussain, 2018; Yang et al., 2021). In this regard, many studies believe that short online reviews often lack a comprehensive evaluation of product characteristics (Ghasemaghaei et al., 2018; Yang et al., 2021). On the contrary, longer online reviews often contain more product details and how and where the product was used in a particular environment, which can reduce the uncertainty of product quality (Mudambi and Schuff, 2012; Zhou and Guo, 2017). Based on the ELM theory, the peripheral cues reflected by review content length will positively influence review helpfulness (Bhattacherjee and Sanford, 2006; Filieri et al., 2018a; Yang et al., 2021). Therefore, based on the extant studies (Mudambi and Schuff, 2012; Zhou and Guo, 2017), we can hypothesize that,

**H1b.** Review content length will positively affect review helpfulness.

### Elaboration likelihood model’s central cue: review label-content relevance

Review label-review relevance refers to the similarity between review labels text and review content. Previous studies found that review text may contain a series of keywords describing attributes from multidimensional dimensions (e.g., product forms, product function, platform service) (Sun et al., 2019; Yang et al., 2021). These review keywords may be related to the contents of the corresponding review labels. Then, customers may make their purchasing decisions based on the relevance between these keywords and the corresponding review labels. Based on the ELM theory, the central cue reflected by review label-content relevance will positively influence review helpfulness (Bhattacherjee and Sanford, 2006; Filieri et al., 2018a; Yang et al., 2021). The higher the relevance of review label-content, the more likely consumers will think that the review is helpful. Thus, it is expected that review label-content relevance will have a positive impact on review helpfulness. Therefore, based on the extant studies (Bhattacherjee and Sanford, 2006; Filieri et al., 2018a; Yang et al., 2021), we hypothesize:

**H2.** Review label-content relevance will positively affect review helpfulness.

### The moderating role of online vs. offline-generated reviews

It is expected that the positive impact of review label volume on review helpfulness will be stronger when the reviews are generated from an omnichannel retailer’s offline channel (Yi et al., 2017). Review label usually reflects a general summarization of the product information described in the review, and the customer can choose whether to continue reading the specific content of the review. In the omnichannel retailing context, the label can serve as highly visible product-based navigation tips (Shami et al., 2011). When the review label is submitted from an omnichannel retailer’s offline channel, the potential readers may regard the label information as more influential than that submitted from the omnichannel retailer’s corresponding online channel (Yang et al., 2019). In other words, the potential readers will have a high level of personal involvement in the offline-based review context than that in the online-based review context. Based on the ELM theory, personal involvement will have significant moderating effects on the impacts of the peripheral cues on review helpfulness (Petty et al., 1981; Yang et al., 2021). Therefore, based on the extant studies (Shami et al., 2011; Yang et al., 2019, 2021), we can hypothesize that,

**H3.** The positive effect of review label volume (H3a) and review content length (H3b) on review helpfulness will be stronger when the review is submitted from an omnichannel retailer’s offline channel.

In the omnichannel retailer’s offline stores, customers can feel, touch, and try products before they purchase them (Huang et al., 2017; Li and Gong, 2022). This suggests that the customers can get more information about the products (Rodríguez-Torrico et al., 2017) where customers have high personal involvement. It is expected that the potential readers will have a high level of personal involvement when they read the reviews submitted from an omnichannel retailer’s offline channel (Yi et al., 2017). Based on the ELM theory, personal involvement will have significant moderating effects on the impacts of the peripheral cues on review helpfulness (Petty et al., 1981; Yang et al., 2021). Indeed, in the omnichannel retailing environment, for reviews submitted from an omnichannel retailer’s offline channel, the label given by the reviewer may not be more consistent with the content of the review. The reason is that reviewers who submit the review from the offline channel will have more motivation and ability to enhance the relevance between the review label and the corresponding review content. On the contrary, for reviews submitted from an omnichannel retailer’s online channel, the review tegs given by the reviewers may not be less consistent with the content of the review than that in the offline-based review context. Therefore, based on the extant studies (Petty et al., 1981; Yang et al., 2021), we can hypothesize that,

**H4.** The positive effect of label-review relevance on review helpfulness will be stronger when the review is submitted from the offline channel.

## Research methodology

### Data collection

To test the proposed research model, empirical data were collected from a famous omnichannel retailer (Suning) in China, which owned more than 3,800 physical stores nationwide (Yang et al., 2019). Suning launched its online store (Suning.com) in January 2010, which ranked among the top three B2C retailers in China. As China’s largest omnichannel retailer, Suning’s online platform included both the traditional reviews generated from customers who purchased the product online and the reviews contributed by customers who purchased the product in its offline stores.

Based on the sampling strategy conducted by Yang et al. (2019), this study selected products that had at least one hundred reviews randomly in six categories of products, namely Air con, Water heater, Range hood, TV, Refrigerator, and Camera. This study employed a Python crawler package to collect the online and offline-based reviews which were filtered and pre-processed to the following fields: review label, review content, review image, review rating, a total of “helpful” votes, and review channels (online-based or offline-based). Some of the online reviews were discarded because of the small amount of review content. Finally, we got 2,822 online reviews of which 1,962 were submitted by an online channel and 860 were submitted by offline stores.

### Measurements

The dependent variable of the proposed model is review helpfulness, which is measured by the vote of review helpfulness. The explanatory variables included review label volume, review content length, and review label-content relevance. Review label volume was calculated by the total number of review labels of the reviews. Review content length was measured by the total number of words in the review text. Review label-content relevance was calculated by the BM25 algorithm. The formula is as follows:


(1) $$S\,c\,o\,r\,e\,\left(q,d\right)=\sum_{i}^{n}W_{i}\,R\,\left(q_{i},d\right)$$

(2) $$I\,D\,F\,\left(q_{i}\right)=\text{log}\,\left(\frac{N-n\,\left(q_{i}\right)+0.5}{n\,\left(q_{i}\right)+0.5}\right)$$

(3) $$R\,\left(q_{i},d\right)=\frac{\left(k_{1}+1\right)\cdot f_{i}\,\left(q_{i},d\right)}{k_{1}\,\left(1-b+b\cdot \frac{L_{d}}{L_{a\,v\,g}}\right)+f_{i}\,\left(q_{i},d\right)}$$

Where *W_i_* represents the weight of the feature, *q_i_*.*R*(*q*_*i*_,*d*)is the correlation score between word *q_i_* and document d. This study utilizes the Robertson-Sparck Jones IDF to represent the weight of the feature *q_i_*.N represents the number of documents. *n*(*q*_*i*_) is the number of documents containing *q_i_*.As for*R*(*q*_*i*_,*d*), K1 and b are adjustment factors, *f_i_* indicates the frequency of *q_i_* in document d. *L*_*d*_is the length of the document and *L*_*avg*_is the average length in document d.

In terms of the moderating variables, the review channel is coded as a dummy variable (1, online channel; 0, offline channel). In terms of the control variables, the review image was measured as the total number of pictures next to the review (Filieri et al., 2018b). Review rating was measured as the number of stars in the review (Mudambi and Schuff, 2012). The review sentiment computation was calculated by using SnowNLP (Chang et al., 2018). SnowNLP is a Python library which is dedicated to analyzing the Chinese language and can deal with text (Wang et al., 2018). We first preprocessed the data to remove some stop words and then applied the SnowNLP to analyze the review text. Finally, we got sentiment scores between –1 and 1 to express text sentiment (Chang et al., 2018; Yang et al., 2021).

### Model specification

We adopted a negative binomial regression to examine how the different variables affect review helpfulness (Chen and Lurie, 2013). Compared with Poisson regression, negative binomial regression can effectively account for omitted variable bias and correct for over-dispersion problems (Haunschild and Beckman, 1998). Thus, we estimated review helpfulness using the following model:


(4) $$Helpfulness=\text{exp}[\beta_{0}+\beta_{1}\left(Image\right)+\beta_{2}\left(\text{Rating}\right)$$

$$+\beta_{3}\,\left(\text{ReviewSentiment}\right)+\beta_{4}\,\left(\text{ReviewLength}\right)$$

$$+\beta_{5}\,\left(\text{LabelVolume}\right)+\beta_{6}\,\left(\mathrm{Label}-\mathrm{Content}\,\mathrm{Relevance}\right)$$

$$+\beta_{7}\,\left(\text{ReviewChannel}\right)+\beta_{8}\,\left(\mathrm{Label}\,\mathrm{volume}\times \text{ReviewChannel}\right)$$

$$+\beta_{9}\,\left(\mathrm{Review}\,\mathrm{length}\times \text{ReviewChannel}\right)$$

$$+\beta_{10}\left(\mathrm{Label}-\mathrm{Content}\mathrm{Relevance}\times \text{ReviewChannel}\right)+\varepsilon ]$$

## Results of model testing

### Results

The descriptive analysis of all the variables and the correlation matrix are shown in Table 1. The correlation coefficients among the main explanatory variables are relatively small, with the maximum being equal to 0.32, which bellowed 0.5. The relatively low correlations between explanatory variables indicate that there is no multicollinearity risk in our models.

**TABLE 1:** Descriptive statistics (*n* = 2,822).

Variables	Min	Max	Mean	SD	1	2	3	4	5	6	7	8
Review helpfulness	0	18	0.07	0.52	1							
Review label volume	1	5	2.60	1.30	0.00	1						
Review content length	1	440	19.31	24.51	0.22	−0.07	1					
Review label-content relevance	−18.21	7.19	−0.85	1.70	0.02	−0.25	−0.05	1				
Online vs Offline	0	1	0.69	0.46	0.00	−0.35	0.14	−0.07	1			
Review Image	0	8	1.47	1.54	0.15	−0.01	0.35	−0.16	0.16	1		
Review Rating	1	5	4.96	0.23	0.01	0.04	0.00	−0.02	−0.03	0.03	1	
Review sentiment	0	1	0.75	0.29	0.00	0.14	−0.18	−0.05	−0.11	−0.00	0.04	1

Table 2 presents the output of the four different models calculated by Negative Binomial Regression. We used Log-likelihood and Pseudo R2 as an assessment of fit. Specifically, model 1 contains control variables. Model 2 contains control variables and explanatory variables. Model 3 contains control variables, explanatory variables, and review channels. Model 4 adds the interaction variables correlated to the review label volume, review content length, and review label-content relevance, respectively. These interaction variables were produced by multiplying the review channels with the explanatory variables. Based on the procedure conducted by Chang and Chuang (2011), before creating the interaction terms, these variables were centralized to avoid multicollinearity.

**TABLE 2:** Negative binomial regression results for review helpfulness.

Variables	Model 1	Model 2	Model 3	Model4
Review Image	0.733***	0.671***	0.680***	0.693***
Review rating	0.879	0.777	0.779	0.716
Review sentiment	0.010	0.519	0.474	0.558
Review label volume		0.122	0.079	0.067
Review content length		0.018***	0.019***	0.016***
Review label-content relevance		0.127*	0.112*	0.219**
Online vs Offline			−0.316	−0.270
Review label volume × Online vs Offline				0.297
Review content length × Online vs Offline				0.017*
Review label-content relevance × Online vs Offline				−0.488*
N	2822	2822	2822	2822
Log likelihood	−580.674	−546.241	−545.407	−538.244
Pseudo R2	0.101	0.154	0.155	0.166
*P* value chi2	0.000	0.000	0.000	0.000

*P < 0.05, **P < 0.01, and ***P < 0.001.

As displayed in Table 2, the impacts of the review label volume on review helpfulness (β = 0.122, *p* > 0.05) were not significant, thus, H1a was not supported. The review content length (β = 0.018, *p* < 0.001) positively affected review helpfulness and validated H1b. In addition, review label-content relevance (β = 0.112, *p* < 0.05) has a positive influence on review helpfulness, thus, H2 was supported.

Furthermore, this study explored the moderating effects of the review channel (online channel vs. offline channel) on the influences of the peripheral path and central path on the helpfulness of review. As shown in the Table 2, the impact of review content length (β = 0.017, *p* < 0.05) on review helpfulness was positively moderated by the review channel. Thus, H3b was supported. However, the impact of review label volume (β = 0.297, *p* > 0.05) on review helpfulness was not significantly moderated by the review channel, thus, H3a was not supported. The impact of review label-content relevance (β = –0.488, *p* < 0.05) on review helpfulness was negatively moderated by the review channel. Thus, H4 was supported. The results of the model test are shown in Table 3.

**TABLE 3:** The results of model test.

Description	Result
H1a. Review label volume will positively affect review helpfulness	Not Supported
H1b. Review content length will positively affect review helpfulness	Supported
H2. review label-content relevance will positively affect review helpfulness	supported
H3a. The positive effect of review label volume on review helpfulness will be stronger when the review submitted from an omnichannel retailers’ offline channel	Not supported
H3a. The positive effect of review content length on review helpfulness will be stronger when the review submitted from an omnichannel retailers’ offline channel	Supported
H4. The positive effect of label-review relevance on review helpfulness will be stronger when the review submitted from the offline channel	Supported

### Robustness checks

To further test the robustness of the statistics results, the present study conducted additional analyses with alternative model specifications. In light of many zero counts with dependent variables, we considered a zero-inflated Negative Binomial Regression model (ZINB) (Yang et al., 2019). Table 4 shows that the result of zero-inflated Negative Binomial Regression is consistent with that of the negative binomial regression.

**TABLE 4:** Robustness check results for alternative model specifications.

Variables	Negative Binomial Regression	ZINB
Review Image	0.693***	0.678***
Review rating	0.716	0.839
Review sentiment	0.558*	0.530
Review label volume	0.067	−0.018
Review content length	0.016***	0.015***
Review label-content Relevance	0.219**	0.229**
Online vs Offline	−0.270	−0.299
Review label volume × Online vs Offline	0.297	0.330
Review content length × Online vs Offline	0.017**	0.016*
Review label-content relevance × Online vs Offline	−0.488*	−0.486*
N	2822	2822

*P < 0.05, **P < 0.01, and ***P < 0.001.

## Discussion and conclusions

### Summary of the findings

Online reviews have attracted many academics and practitioners’ attention as it has become a commonly used tool for customers to make purchase decisions. Extant studies mainly focus on the factors that affect review helpfulness in a single channel. The factors that influence review helpfulness in an omnichannel retailing environment are still unexplored. Based on the Elaboration Likelihood Model, a research model was developed and empirically tested based on the data of 2,822 product reviews collected from Suning.com. This study obtained several interesting and important findings.

First, the present study found that review content length positively influences review helpfulness, which is consistent with the extant studies (Chua and Banerjee, 2015; Hong et al., 2017; Yang et al., 2021). This suggests that the helpfulness of a review will increase when there are more words in a review in the omnichannel retailing context. This study found that review label-content relevance has a positive impact on review helpfulness in the omnichannel retailing environment. This suggests that the relationship between review label and review content plays an important role in shaping readers’ perception of the helpfulness of the review. However, this study found that the impacts of review label volume on review helpfulness in the omnichannel retailing context were not significant, which is different from our hypothesis, H1a. The possible explanation is that the Suning.com website contains some review labels for reviewers and readers. These review labels or tags can be regarded as the extraction and integration of online reviews. Reviews with high relevance of review labels may be more authentic and reliable, while the number of review labels only simply indicates that the reviews contain a large amount of information, but are unable to reflect its authenticity.

Second, this study found that the impact of review content length on review helpfulness was significantly moderated by the review channel. This suggests that the positive effect of review content length on review helpfulness will be stronger when the reviews are submitted from the omnichannel retailer’s online channel. The possible explanation is that in the offline channel, customers may have a deep understanding of the product with the help of other customers and sales staff, while in the online channel, customers only have online shopping experiences without the experiences of the omnichannel retailer’s offline stores, which will make customers not know the quality of the product. Thus, these online-based reviews may contain the quality of the product and the customer’s feelings. As a result, online reviews generated from the online channel are usually longer than those from the offline channel. In addition, the influence of review label-content relevance on review helpfulness is negatively moderated by the review channel. This suggests that the positive effect of review label-content relevance on review helpfulness will be stronger when the review is submitted from the omnichannel retailer’s offline channel. The possible explanation is that the review submitted from the omnichannel retailer’s offline channel is generated by customers who had experienced the product features and seller’s service in the corresponding physical store. Thus, the review labels of these offline-based reviews are more likely to be consistent with the specific situation of the product, which further helps to improve the review helpfulness.

In terms of control variables, our findings demonstrate that review rating has no significant effect on review helpfulness, which is consistent with the findings of De Pelsmacker et al. (2018). This study found that the influences of review sentiment on review helpfulness were not significant. This is different from previous studies (Chua and Banerjee, 2016; Malik and Hussain, 2017), which reported that review sentiment has a significant impact on review helpfulness. The possible explanation is that there are two different types of reviews in the omnichannel retailing environment, namely online-generated reviews and offline-generated reviews. These two different reviews’ content sentiment may sometimes have a complementary sentiment model. Moreover, the present study found that the number of review images has a significant effect on review helpfulness. This is consistent with previous research (Karimi and Wang, 2017). This suggests that the review images provided by reviewers may reveal the most important information about the product or service. Thus, the more review images submitted, the more likely consumers may regard the review to be helpful.

### Theoretical implications

The present study has several theoretical implications for the literature. First, different from many extant studies focused on the antecedents of review helpfulness in a single online channel environment (Filieri et al., 2017; Yang et al., 2021), our study explains factors that affect review helpfulness in an emerging omnichannel retailing context. Specifically, the present study validated how central cue (review label-content relevance) and peripheral cue (review content length and review label volume) affect the review helpfulness in the omnichannel retailing context by including both online-based and offline-based reviews.

Second, our study applied the ELM theory in the omnichannel retailing environment and validated it as a suitable theoretical foundation to explain and predict review helpfulness in the emerging retailing context. Our study confirms that review channels have a significant moderating effect on the two routes for review helpfulness formation. This study, thus, provides important insights on how to review channel may shape review helpfulness in an omnichannel shopping context.

### Practical implications

This study has several practical implications. First, omnichannel retailers should realize the important influence of review content length on review helpfulness. The implication for the omnichannel retailers is that they could appropriately extend the limit on the number of review words, or set a minimum number of words in their review systems, because longer reviews may contain more details about product quality and after-sales service.

Second, the omnichannel retailers should pay attention to the relevance of review labels and review content in that the present study validated the positive influences of review label-content relevance on review helpfulness. The implication for omnichannel retailers is that they should improve their ability to review text analysis and enhance the consistency between the information contained in online reviews and review labels to better capture the main information of the reviews.

Third, the present study also found that the impacts of review content length and review label-content relevance on review helpfulness were different for reviews submitted from the omnichannel retailer’s different shopping channels. The implication for omnichannel retailers is that they should pay more attention to the reviews generated from their offline stores because the effect of review label-content relevance on review helpfulness will be stronger when the reviews are submitted from the omnichannel retailer’s offline channel.

### Conclusion

Based on the ELM theory, the present study examined the factors that affect review helpfulness in the omnichannel retailing context. A research model reflecting central cue (review label-content relevance) and peripheral cue (review content length, review label volume) on review helpfulness has developed. The research model empirical examined based on data collected from 2,822 product reviews of Suning.com. The results of Negative Binomial Regression indicated that review label-content relevance and review content length positively influence review helpfulness. In addition, the review channel has a positive moderating effect on the relationship between review content length and review helpfulness but a negative moderating effect on the association between review label-content relevance and review helpfulness.

The results of the present study should be interpreted by considering its limitations. First, due to the common drawback of the research that collected secondary data online, this study only includes review posters. The opinions from these non-responding customers who have shopping experience in an omnichannel retailer’s online or offline stores did not analyze in the present study. Future studies are encouraged to employ other complementary research methods, such as surveys or experiments to examine the issue of review helpfulness in the omnichannel retailing context. Second, the data used in the present study were collected from a specific omnichannel retailer’s website—Suning.com, China. Future studies thus are encouraged to retest our research model by collecting the data from different omnichannel retailers in other countries to validate the generalizability of the present study. Finally, as our main research purpose is to examine the factors that affect review helpfulness in the omnichannel retailing context, we did not examine the impacts of factors, such as consumer engagement (Kliestik et al., 2022b), expectations (Hopkins, 2022), purchasing habit (Watson, 2022), and behavioral intentions (Kliestik et al., 2022a) on review helpfulness. Therefore, future studies are encouraged to explore the effects of these factors on review helpfulness in the omnichannel retailing environment.

## Data availability statement

The raw data supporting the conclusions of this article will be made available by the authors, without undue reservation.

## Author contributions

ZZ: conceptualization and writing-original draft preparation. HJ: supervision, writing-reviewing, and editing. CZ: data curation and investigation. JZ: data curation and editing. SY: supervision and methodology. All authors contributed to the article and approved the submitted version.
